# Symptoms of delusion: the effects of discontinuation of low-dose venlafaxine

**DOI:** 10.1111/j.1600-0447.2009.01433.x

**Published:** 2009-10

**Authors:** M Koga, F Kodaka, H Miyata, K Nakayama

**Affiliations:** 1 Department of Psychiatry, Machida Municipal HospitalTokyo, Japan; 2 Department of Psychiatry, Sobu HospitalChiba, Japan; 3 Department of Psychiatry, Jikei University School of MedicineTokyo, Japan

**Keywords:** antidepressives, antipsychotics, depression, psychopharmacology, side effects

## Abstract

**Objective:**

We report a patient who experienced delusional symptoms during gradual discontinuation of low-dose venlafaxine and required antipsychotic treatment.

**Method:**

Case report.

**Results:**

A 31-year-old woman with major depression had been treated abroad with venlafaxine before returning to Japan. Since venlafaxine is unavailable here, we supplemented her regular venlafaxine dosage of 37.5 mg/day with clomipramine 20 mg/day. After 5 weeks we reduced venlafaxine to 18.75 mg/day and uptitrated clomipramine to 40 mg/day. Four days later she developed delusions of reference, palpitations and nausea. Clomipramine was increased to 60 mg/day, and her symptoms subsided. Eight weeks later her supply of venlafaxine ran out, and within 4 days her condition deteriorated into more severe symptoms that required 4 months’ antipsychotic treatment.

**Conclusion:**

We speculate that her symptoms were discontinuation syndrome, including psychotic symptoms and physical symptoms, caused by (i) venlafaxine-clomipramine interaction and/or (ii) the serotonin reuptake inhibitor-like effects of low-dose venlafaxine.

## Introduction

Venlafaxine, a serotonin-norepinephrine reuptake inhibitor, is known to carry a risk of discontinuation syndrome due to its relatively short half-life. The frequency of discontinuation syndrome was reported to increase with higher doses and abrupt discontinuation. The common symptoms are physical, including dizziness, headaches and nausea, as well as psychiatric symptoms such as agitation and anxiety (1, 2). Conversely, reports of psychotic symptoms have been few (2). We report a patient who experienced psychotic and physical symptoms during gradual discontinuation of low-dose venlafaxine while being administered clomipramine. That combination of symptoms fits the diagnostic criteria for discontinuation syndrome and required long-term antipsychotic treatment until resolution.

## Case report

A 31-year-old woman with major depression came to our out-patient clinic after having lived abroad. She had no prior history of physical illness, delusional symptoms, manic episodes or substance abuse. Before moving abroad, she had suffered from mild depressive mood, slight anxiety and low self-confidence for the first time, and had been treated for about 1 year with clomipramine (20–40 mg/day).She fully recovered and safely stopped medication (i.e. she experienced no discontinuation syndrome) 2 months before leaving Japan. Within 6 months after going abroad, she had a recurrence, was treated with venlafaxine for 6 months and almost recovered on a dosage of 37.5 mg/day before returning to Japan. When we first saw her she exhibited a slight depressive mood. Since venlafaxine is not available in Japan, we decided to start her on another antidepressant and gradually discontinue venlafaxine. We began coadministration of 20 mg/day of clomipramine, which she had previously taken and had been safe and effective for her condition, and her regular venlafaxine dosage of 37.5 mg/day. Her symptoms subsided after 5 weeks, so we reduced venlafaxine from 37.5 to 18.75 mg/day and uptitrated clomipramine from 20 to 40 mg/day. Since the imipramine equivalent doses of venlafaxine and clomipramine are the same (3), the decrease in the venlafaxine dosage was almost exactly compensated by the increase in the clomipramine dosage. Unfortunately, 4 days later, she began to suffer from depressive mood and delusions of reference along with palpitations and nausea. Therefore, we increased the clomipramine to 60 mg/day. Two weeks later her depressive mood and physical symptoms had recovered, but she still exhibited some slight indications of delusional feelings.

The patient’s condition remained stable until her supply of venlafaxine ran out 8 weeks later, and deterioration was seen within 4 days. She lapsed back into delusions of persecution and fear of death along with palpitations, dizziness, nausea and stomachache that rendered her unable to work. At this stage, because we were unable to resume venlafaxine, we decided to administer an antipsychotic agent, perospirone, which is a type of serotonin dopamine antagonist available only in Japan (4). The dosage was 8 mg/day at the beginning. After 5 weeks her symptoms began to show visible improvement. It took a further 3 months of this antipsychotic treatment for her to gradually recover from her symptoms and be able to work in the same capacity as before. Meanwhile, perospirone was tapered down to 4 mg/day and finally 2 mg/day in the last month and discontinued. Twelve months have now passed since then, and the patient has completely recovered and shows no signs of any depressive mood or delusional symptoms on the current clomipramine dosage of 60 mg/day.

## Discussion

Our case is characterized by several significant findings. First, the patient exhibited prolonged delusional symptoms and fear of death as well as palpitations and nausea after discontinuation of venlafaxine. Her condition was diagnosed as venlafaxine discontinuation syndrome because she experienced not only psychotic symptoms but also physical symptoms (5). Second, she experienced these symptoms during gradual discontinuation of an extremely low dosage of venlafaxine combined with clomipramine, and they finally required long-term antipsychotic treatment. We speculate that the following factors contributed to the manifestation of the symptoms in this patient.

First, the symptoms may have resulted from an interaction between venlafaxine remaining in her system and clomipramine. *In vitro* studies indicate that venlafaxine is a relatively weak inhibitor of cytochrome P450 (CYP) 2D6 (1), and clomipramine is metabolized by CYP 1A2, 2C, 2D6 and 3A4 (6). Therefore, some changes in clomipramine metabolism may have been caused by adding or stopping venlafaxine, leading to some adverse reactions. A study of eight patients by Gomez and Perramon showed that, when venlafaxine was added to clomipramine, none of the patients experienced any adverse reactions (one showed an increase in the serum clomipramine level, but the others did not) (7). Meanwhile, Benazzi reported a patient who developed severe anticholinergic adverse reactions and hand tremors when clomipramine was augmented with venlafaxine (data on the serum clomipramine level were not reported) (8). Another patient experienced no adverse reactions and no change in the serum clomipramine level upon stopping venlafaxine (8). Although the data in those case studies were somewhat limited and we were unable to determine our patient’s serum clomipramine level, we speculate that there might have been an interaction between the two drugs in our patient. Furthermore, the sensitivity to adverse reactions to drugs differs among individuals.

Another possibility may be that our patient had intolerance to the discontinuation of low-dose venlafaxine. She experienced psychotic and physical symptoms not only when the dosage of venlafaxine was decreased from 37.5 mg/day to 18.75 mg/day, but also when 18.75 mg/day of venlafaxine was discontinued. Clomipramine was being coadministered with venlafaxine at both times, but the clomipramine dose remained unchanged in the second case. Given the stable dosage of clomipramine when venlafaxine was discontinued, it would seem that the symptoms that occurred were a result of the gradual discontinuation of the low-dose venlafaxine, although, as noted above, there may have been interaction between venlafaxine remaining in her system and clomipramine. It was reported that patients generally experience discontinuation syndrome upon stopping higher dosages of venlafaxine (2). However, there have been reports that in discontinuation syndrome associated with low-dose venlafaxine certain patients developed severe psychotic symptoms, as was the case with our patient. Louie et al. (9) reported a 46-year-old woman who experienced auditory hallucinations 3 days after reducing the venlafaxine dosage (from 37.5 mg b.i.d. for 10 days to 18.75 mg b.i.d.). The symptoms persisted until her venlafaxine dosage was increased to 56.25 mg/day. Regarding combination therapy, Parker and Blennerhassett (10) reported a 42-year-old man who had auditory hallucinations for a period of 5 days after discontinuing treatment with 75-mg/day venlafaxine combined with 1000 mg/day lithium. Fava (11) described a 59-year-old man with bipolar disorder (type 1) who manifested manic symptoms after venlafaxine (37.5 mg/day) was discontinued and replaced with lithium. The symptoms resolved within a few days after venlafaxine was restarted at 37.5 mg/day. Venlafaxine was discontinued after 3 months, and the patient again experienced discontinuation symptoms, which subsided within 2 weeks. All of the above patients also experienced physical symptoms.

We believe that discontinuation of the low dosage of venlafaxine played a significant role in these symptoms. One possible explanation for these symptoms is the serotonin reuptake inhibitor (SRI)-like effects of venlafaxine at low dosages. It was reported that, like other SRIs, venlafaxine selectively inhibited 5-HT uptake at low dosages (12), while others reported that discontinuation of SRIs resulted in a rapid decrease in serotonin availability in the brain (13). This accounts for the manifestation not only of physical symptoms such as dizziness and intestinal symptoms, but also of psychiatric symptoms. It is likely that the same mechanism as in SRI discontinuation occurs in discontinuation of low-dose venlafaxine and induces these unusual psychotic and physical symptoms.

Drug manufacturers recommend gradual reduction of the dose of venlafaxine to prevent discontinuation syndrome. However, our patient shows that unusual psychotic and physical symptoms (i.e. discontinuation syndrome) can be associated even with discontinuation of a low dose of venlafaxine, even when the discontinuation is gradual, and even when combined with other drugs. It is important, therefore, to consider that some patients may show intolerance to even low-dose venlafaxine discontinuation.
